# Identifying the barriers of smoking cessation and predictors of nicotine dependence among adult Malaysian smokers: A cross-sectional study

**DOI:** 10.18332/tid/154964

**Published:** 2022-12-07

**Authors:** Roy R. Marzo, Kareem A. El-Fass, Nermin A. Osman, Thin M. Kyaw, Prem Kumar A/L Arivanandan, Logithasan Murale Morgan, Keiswini Latchumana, Kirthanashree Arasu, Abiodun D. Obaromi, Yulan Lin

**Affiliations:** 1 Department of Community Medicine, International Medical School, Management and Science University, Shah Alam, Malaysia; 2 Department of Community Medicine, Faculty of Medicine, Asia Metropolitan University, Bandar Seri Alam, Malaysia; 3 Global Public Health, Jeffrey Cheah School of Medicine and Health Sciences, Monash University Malaysia, Kuala Lumpur, Malaysia; 4 Department of Pharmacy Practice, College of Clinical Pharmacy, King Faisal University, Hofuf, Egypt; 5 Department of Biomedical Informatics and Medical Statistics, Medical Research Institute, Alexandria University, Alexandria, Egypt; 6 Department of Community Medicine, Faculty of Medicine, University of Cyberjaya, Cyberjaya, Malaysia; 7 Department of Community Medicine, Faculty of Medicine, Manipal University College Malaysia, Melaka, Malaysia; 8 Department of Biostatistics, Federal University of Health Sciences, Otukpo, Nigeria; 9 Department of Epidemiology and Health Statistics, School of Public Health, Fujian Medical University, Fuzhou, People's Republic of China

**Keywords:** smoking cessation, nicotine dependence, barriers, sociodemographic status

## Abstract

**INTRODUCTION:**

Proper understanding of the prevalence and determinants of nicotine dependence is crucial for developing and implementing effective tobacco control interventions. The aim of the study was to identify the intrinsic and extrinsic barriers to smoking cessation, and to assess the association between nicotine dependence with demographic variables in Malaysia.

**METHODS:**

A cross-sectional survey based on the Challenges to Stopping Smoking Scale (CSS-21) and Fagerström test for nicotine dependence (FTND) was performed on smoking Malaysian citizens aged ≥18 years, from February to June 2021.

**RESULTS:**

A total of 1026 parents responded to the survey. As for the smoking dependence based on FTND, 39.1% suffered low-moderate dependence, while about 33.6% suffered moderate dependence. Only 1.8% suffered high dependence. Considering the barriers of quitting smoking based on CSS-21, the mean score of the intrinsic barriers domain was 5.7 ± 2.9, and for the extrinsic domain was 7.4 ± 4.0. The most common barrier reported in the intrinsic domain was the easy availability of cigarettes (69.8%), followed by experiencing withdrawal symptoms (68.5%). On the other hand, the most common barrier reported in the extrinsic domain was the belief in the capability of stopping smoking in the future (72.8%), followed by the fear of having side effects after stopping smoking (63.2%). Gender, race, education level, occupation, marital status, place of residence, and monthly income were also significantly associated with the FTND nicotine dependence category (all p<0.05). Pearson correlation analysis reported a positive association between intrinsic score (r=0.38), extrinsic score (r=0.43) and FTND score (all p<0.001).

**CONCLUSIONS:**

Barriers to stopping smoking should be taken into consideration in initiatives to decrease smoking-related mortality. Vulnerable populations that are susceptible to high nicotine dependence should be given particular attention.

## INTRODUCTION

Smoke is a complex hazardous mixture of chemicals and additives, produced by burning tobacco. Tobacco smoke contains nicotine and monoamine oxidase (MAO) inhibitor which results in addictive and euphoriant properties^1^. Nicotine is an alkaloid that can be found abundantly in the tobacco plant and it is classified as a central nervous system stimulant drug^2^. Nicotine dependence is well known as a common preventable cause of death around the globe. However, the World Health Organization (WHO) estimates that the number of smokers is expected to increase from 1.3 billion to 1.7 billion worldwide by the year 2025^3^, with particularly serious health impacts in developing countries^4^. Malaysia is a multi-ethnic country and has not been spared from this pandemic, every year 10000 nicotine-dependent related deaths are registered^5^, and smoking-related diseases have been listed as the main cause of disability-adjusted life years and lost years of life among the Malaysian population^6^.

A proper understanding of the prevalence and determinants of nicotine dependence is crucial for developing and implementing effective tobacco control interventions. Studies have identified that individual-level socioeconomic status (SES) factors like age, sex, marital status, income, educational level, and other occupational factors are the main determinants of nicotine dependence^7^. It was found that males with a low level of education and income are suffering from higher nicotine dependence. Thus, identifying subgroups of youth who may be at greater risk than others to develop nicotine addiction is an important step forward in preventing smoking initiation and controlling tobacco use^8^. Furthermore, little evidence exists on the influence of different regulatory national approaches on nicotine dependence. Among adults, primary evidence suggests that more restrictive measures could yield a lower prevalence of smoking. Despite of significant hypothetical expectations, it was found that there is a low compliance rate prominently seen in the communities^9^. Still, there is a debate around the relationship between contextual SES and nicotine dependence.

Stopping smoking usually involves a premeditation not to smoke any more cigarettes from a given point of time, followed by self-conscious resistance resulting in a period of abstinence. Most of the studies conducted in Malaysia were small sized cross-sectional survey and directed toward factors that contribute to smoking initiation in adolescents. Nonetheless, there are limited studies conducted to study the barriers beyond the smoking cessation. Thus, the aim of the study was to identify the barriers of smoking cessation, and to assess the association between nicotine dependence with individual sociodemographic status among adult smokers in Malaysia.

## METHODS

### Study design and participants

This cross-sectional study was conducted in Malaysia from February to June 2021. The target participants were smoking Malaysian citizens aged ≥18 years who could read and understand Bahasa Melayu or English. Due to the limitations in employing face-to-face methods during the outbreak, the survey was prepared in a Google form and disseminated to the participants using a snowball sampling method. First, we recruited 50 primary participants and asked them to share the questionnaire link with individuals in their social network who met the inclusion criteria. We chose these social media platforms because they are widely used among the population across sociodemographic characteristics. The sample size was calculated via the Raosoft sample size calculator. The sample size was calculated at a 95% significance level and a 5% margin of error, population proportion of 50% of 32.7 million residents of Malaysia, so the representative sample size was 385 participants. In order to account for 20% non-respondents, the total sample size was 462 participants.

### Data collection procedures

We distributed the questionnaire using personal contacts using word of mouth or emails and through web-based applications and social media such as Facebook, Instagram, LinkedIn, Telegram, Twitter, and WhatsApp. Participants were reminded to respond only once. We employed unique identifiers for use only in a single account by settings that allow only one response per user. Participants were assured the confidentiality and privacy of their responses, to reduce potential bias introduced by self-reported data.

### Instruments

Challenges to stopping smoking scale (CSS-21) was developed by Thomas et al.^10^ to determine the challenges or problems associated with stopping smoking^10^. It has a total of two subscales and assesses items as follows: 9 items related to the personal aspect of quitting (e.g. ‘feeling lost without cigarettes’), and 12 items related to social or environmental aspects of quitting (e.g. ‘fear of failing to stop smoking’). The first subscale is labeled as ‘intrinsic factors’ (items 1–9) and the second subscale as ‘extrinsic factors’ (items 10–21). It consists of 21-items scored on a 4-point Likert-type scale. Participants’ responses to each item vary from 1 (not a challenge) to 4 (major challenge). In scoring the scale each subscale is assessed separately. The total scale score is not calculated. The average score of a subscale is obtained by summing up the scores the individual gets from each item in that subscale. The scores of the ‘intrinsic scale’ range 9–36 points, and the ‘extrinsic scale’ range 12–43 points. A higher score indicates greater challenges. The original Cronbach’s alpha values of the CSS-21 subscales were 0.86 to 0.82, respectively10. Uysal et al.^11^ adapted into Turkish the FTND^12^ , with which we compare the consistency of our results. The scale contains six items that evaluate an individual smoker’s nicotine dependence. Based on the Turkish validity and reliability study, the internal consistency of the scale was 0.56^11^. In this study, the Cronbach’s alpha coefficient was calculated as 0.76.

### Statistical analysis

The data are described as mean and standard deviation (SD) for numerical variables, and frequency and percentage for categorical variables. Chi-squared test was used to test for the associations between categorical variables, while Pearson correlation was used to test for the correlation of numerical variables. The significance level is set at 0.05. The statistical analysis was conducted using IBM SPSS Statistics for Windows, Version 25.0 (IBM Corp. Released 2017 Armonk, NY: IBM Corp.).

Responses for the intrinsic and extrinsic domains of the CSS-21 questions were coded as 1 for ‘Yes’ and 0 for ‘No’ to calculate the scores. The full scores are 9 and 12, for the intrinsic and the extrinsic domain, respectively. The same coding was applied for the responses of the FTND except for questions 1 and 4 of the FTND; they were coded 0 to 3 ascendingly according to the dependence. Nicotine dependence was categorized according to the FTND score as follows: 0–2 as low; 3–4 as low-moderate, 5–7 as moderate, and ≥8 as high.

## RESULTS

A total of 1026 participants responded to the survey. The mean age of the respondents was 34.4 ± 12.0. Most of the respondents were males (81.5%), and Indian (42.0%). The responses came from people of varying education level, where most had the highest level as secondary (39.0%) and post-secondary (29.6%). Most participants were employed fulltime (51.7%) with monthly income of less than 4849 RM (75.0%) and married (54.0%). Most of the respondents lived in urban areas (74.2%). The full results of the sociodemographics are presented in Table 1. Regarding the smoking habits of the study participants (Table 2), about 45.5% were old smokers and about 52.0% started smoking at an age of 18–34 years. They smoked mostly daily (50.6%). On the other hand, about 54.1% of them reported they have been smoking for <5 years. As for the smoking dependence of our respondents (Table 3), 39.1% had low-moderate dependence, while about 33.6% moderate dependence, and only 1.8% had high dependence.

**Table 1:** Characteristics of the study population, Malaysia, 2021 (N=1026)

*Demographics*	*n (%)*
**Age** (years), mean ± SD	34.4 ± 12.0
**Gender**	
Male	836 (81.5)
Female	190 (18.5)
**Race**	
Indian	431 (42)
Malay	339 (33)
Chinese	178 (17.4)
Other	78 (7.6)
**Educational level**	
No formal	60 (5.8)
Primary	54 (5.3)
Secondary	400 (39)
Tertiary post-secondary	208 (20.3)
Pre-university	304 (29.6)
**Employment status**	
Unemployed	69 (6.7)
Full-time	531 (51.7)
Part-time	253 (24.7)
Student	173 (16.9)
**Marital status**	
Single	320 (31.2)
Married	554 (54)
Divorced	109 (10.6)
Widowed	25 (2.4)
Other (Engaged)	18 (1.8)
**Residence**	
Urban	761 (74.2)
Rural	265 (25.8)
**Monthly income (RM)**	
≤4849	769 (75.0)
4850–10959	237 (23.1)
≥10960	20 (1.9)

RM: 100 Malaysian Ringgit about 120 US$.

**Table 2:** Smoking status of respondents, Malaysia, 2021 (N=1026)

*Characteristics*	*n (%)*
**Smoking status**	
Never smoker	146 (14.2)
Old smoker	467 (45.5)
New smoker	365 (35.6)
Quitter	48 (4.7)
**Frequency of smoking**	
Not at all	182 (17.7)
Less than daily	325 (31.7)
Daily	519 (50.6)
**Duration of smoking** ( years )	
<1	266 (25.9)
1–5	555 ( 54.1)
>5 to <10	127 (12.4)
10	20 (1.9)
>10	58 (5.7)
**Number of cigarettes per day**	
≤ 10	800 (78)
11–20	173 (16.9)
>20	53 (5.2)
**Age of starting smoking (years)**	
<18	354 (34.5)
18–34	534 (52.0)
35–44	95 (9.3)
45–54	25 (2.4)
55–64	9 (0.9)
≥65	9 (0.9)

**Table 3:** Smoking dependence of respondents represented as Fagerström test for nicotine dependence (FTND) categories, Malaysia, 2021 (N=1026)

*FTND categories*	*n (%)*
Low	262 (25.5)
Low-moderate	401 (39.1)
Moderate	345 (33.6)
High	18 (1.8)

Considering the barriers of quitting smoking, the mean score of the intrinsic barrier’s domain was 5.7 ± 2.9, and for the extrinsic domain is 7.4 ± 4.0. The most common barrier reported in the intrinsic domain was the easy availability to cigarettes (69.8%), followed by experiencing withdrawal symptoms (68.5%). On the other hand, the most common barrier reported in the extrinsic domain was the belief of capability of stopping smoking in the future (72.8%), followed by the fear of having side effects after stopping smoking (63.2%). The frequency and percentage of the individual questions of each domain are presented in Table 4.

**Table 4:** Responses to intrinsic and extrinsic barriers question, Malaysia, 2021

	*n (%)*
**Intrinsic barriers**	
Have you experienced any of the following withdrawal symptoms: depression, anxiety, restlessness, irritability, sleeplessness, craving?	703 (68.5)
Have you felt lost without cigarettes?	631 (61.5)
Are you addicted to cigarettes?	596 (58.1)
Have you experienced any mixed emotions such as anger or feeling upset when you were trying to stop smoking?	657 (64.0)
Have you experienced any stressful event when you were trying to stop smoking?	618 (60.2)
Have you thought about never been able to smoke again after you stopped smoking?	640 (62.4)
Have you felt bored when you were trying to stop smoking?	635 (62.0)
Do you see things or people that reminded you of smoking?	649 (63.3)
Are cigarettes easily available to you?	716 (69.8)
**Extrinsic barriers**	
Have you faced any difficulties in finding someone to help you stop smoking?	624 (61.0)
Have you felt a lack of support or encouragement from health professionals to stop smoking?	647 (63.0)
Have the expensive prices of stop-smoking medicines (nicotine replacement therapy) stopped you from quitting?	621 (60.5)
Do you fear having side effects from stop-smoking medicines?	648 (63.2)
Have you felt a lack of encouragement or help from family or friends to stop smoking?	616 (60.0)
Do you fear gaining weight if you stop smoking?	607 (59.2)
Did any family members or friends encourage you to smoke?	584 (56.9)
Do you fear failing to stop smoking?	610 (59.5)
Do you believe that stop-smoking medicines do not work?	639 (62.3)
Do you fear quitting smoking may interrupt social relationships?	605 (59.0)
Do you believe that you can stop smoking in the future, if you need to?	747 (72.8)
Do you use any other substances such as cannabis or alcohol?	640 (62.4)

There was a statistically significant association between the FTND categories of smoking dependence and the sociodemographics of the participants, as presented in Table 5. For those with low dependence, the majority were Indian (70.0%), with tertiary education (41.6%), students (36.6%), and single (57.1%). On the other hand, those suffering low-moderate dependence were mostly Indian (44.9%), with secondary education (39.1%), employed full-time (49.3%), and married (54.4%). Participants suffering moderate dependence were mostly Malay (53.9%), with secondary education (53.9%), also employed full-time (65.8%), and married (70.4%). Respondents suffering high dependence were mostly Malay (50.0%), with post-secondary education (33.3%), employed full-time (72.2%), and married (38.9%). Urban residence and monthly income less than 4849 RM were most common among all the dependence groups.

**Table 5:** Associations between sociodemographic factors and nicotine dependence (represented by FTND categories), Malaysia, 2021 (N=1026)

*Factors*	*FTND categories*				p*
	*Low (n=262) n (%)*	*Low-moderate (n=401) n (%)*	*Moderate (n=345) n (%)*	*High (n=18) n (%)*	
**Gender**					<0.001
Male	190 (72.5)	337 (84.0)	293 (85.0)	16 (89.0)	
Female	72 (27.5)	64 (16.0)	52 (15.0)	2 (11.0)	
**Race**					<0.001
Malay	39 (15.0)	105 (26.2)	186 (53.9)	9 (50.0)	
Chinese	33 (12.6)	88 (21.9)	52 (15.1)	5 (27.8)	
Indian	184 (70.0)	180 (44.9)	64 (18.5)	3 (16.7)	
Other	6 (2.4)	28 (7.0)	43 (12.5)	1 (5.5)	
**Education level**					<0.001
No formal	4 (1.5)	25 (6.2)	27 (7.8)	4 (22.2)	
Primary	7 (2.7)	26 (6.5)	20 (5.8)	1 (5.6)	
Secondary	55 (21.0)	157 (39.1)	186 (53.9)	2 (11.1)	
Tertiary	109 (41.6)	60 (15.0)	34 (9.9)	5 (27.8)	
Post-secondary/Pre-university	87 (33.2)	133 (33.2)	78 (22.6)	6 (33.3)	
**Employment status**					<0.001
Unemployed	16 (6.1)	26 (6.5)	25 (7.2)	2 (11.1)	
Full-time	93 (35.5)	198 (49.3)	227 (65.8)	13 (72.2)	
Part-time	57 (21.8)	125 (31.2)	68 (19.8)	3 ( 16.7)	
Student	96 (36.6)	52 (13.0)	25 (7.2)	0 (0)	
**Marital status**					<0.001
Single	149 (57.1)	120 (30.0)	48 (14.0)	3 (16.7)	
Married	86 (32.8)	218 (54.4)	243 (70.4)	7 (38.9)	
Divorced	20 (7.5)	48 (11.9)	36 (10.4)	5 (27.8)	
Widowed	4 (1.5)	7 (1.7)	13 (3.8)	1 (5.5)	
Engaged	3 (1.1)	8 (2.0)	5 (1.4)	2 (11.1)	
**Residence**					<0.001
Urban	213 (81.3)	256 (63.8)	280 (81.2)	12 (66.7)	
Rural	49 (18.7)	145 (36.2)	65 (18.8)	6 (33.3)	
**Monthly income** (RM)					<0.001
≤4849	207 (79.0)	322 (80.0)	228 (66.0)	12 (66.7)	
4850–10959	46 (17.6)	72 (18.0)	113 (33.0)	6 (33.3)	
≥10960	9 (3.4)	7 (2.0)	4 (1.0)	0 (0)	

* Chi-squared test. RM: 100 Malaysian Ringgit about 120 US$.

Considering the barriers to smoking quitting, there was a moderate positive association between the FTND score and the intrinsic domain score (r=0.38; p<0.001), and the extrinsic domain sore (r=0.43; p<0.001) as per the Pearson correlation coefficient.

## DISCUSSION

The common core elements widely used to measure SES are education level, income, marital status, and employment, and their influence on smoking habit in different communities. Furthermore, recent approaches have been developed to emphasize the intrinsic and extrinsic barriers of smoking cessation using the CSS-21 questionnaire. However, there is abundant literature studying the role of SES in tobacco use, yet many aspects of this relationship remain underexplored. The results augment the popular phenomenon that smoking and nicotine dependence are highly prevalent among men in Malaysia. There was a high prevalence among participants from the urban regions and of Indian race. It was found that individual education level was inversely associated with the probability of nicotine dependence, with a high prevalence among those with a secondary school education. Moreover, our data indicate that full-time employment was associated with a high rate of cigarette consumption. Our findings are coherent with the results found in other Asian and Western studies^13-16^.

In the current study, higher individual monthly income showed a significant negative association with nicotine dependence. In terms of the barriers of quitting smoking, the most frequent intrinsic barriers were the easy availability to cigarettes (69.8%), and experiencing withdrawal symptoms (68.5%). On the other hand, the most frequent barriers reported were the belief of capability of stopping smoking in the future (72.8%), and the fear of having side effects after stopping smoking (63.2%). These findings are consistent with the results from some western countries^17-19^, however they differed from a previous Chinese study^16^, where individuals with lower income are more likely to be nicotine addicted. Perhaps individuals with higher income are more likely to afford tobacco products and therefore consume more. Living in communities with higher overall education level and good income was associated with a decreased risk of nicotine dependence^20^. This may be because the people with higher education level tend to have a healthier lifestyle. The strong association between both contextual and individual education level and risk of nicotine addiction in our study suggests that implementing robust smoking cessation programs to reduce tobacco use in Malaysia should focus on the less educated in parallel with those living in communities with low levels of education^21^.

### Limitations

The current study has some limitations. First, it is based on self-reported questionnaire, and may therefore be liable for recall bias. Second, the effects of SES on smoking dose or levels of tobacco consumption have been more challenging to be explored because SES is multifactorial construct that cannot be directly measured by a single indicator. Third, our results revealed a significant correlation between the intrinsic and extrinsic barrier scores, however, it is not deeply analyzed using regression modeling. Moreover, due to the ongoing COVID-19 pandemic, a snowballing technique was applied for sampling which allowed us to assess the intrinsic and extrinsic barriers to cessation, but did not provide generalizable results of prevalence, which was not however the aim of the study.

### Recommendations

Interventions to remove the general public’s both intrinsic and extrinsic barriers are needed. Implementing strategies as top priorities to improve self-efficacy to quit smoking and enhance the support of health professionals to target smoker populations. The government should establish more restrictive measures to limit cigarette availability, while providing more support for smokers to overcome withdrawal symptoms. Particular attention should also focus on vulnerable populations to high nicotine dependence.

## CONCLUSIONS

This study depicts a high prevalence of smoking and nicotine dependence and low level of attempting to quit smoking among current smokers in Malaysia. Additionally, nicotine dependence varies by both contextual and individual SES variables. These findings highlight the need for tobacco cessation interventions to target men who are fully employed, less educated, and from poorer communities.

## Data Availability

The data supporting this research are available from the authors on reasonable request.
